# Supplementary material for: Instant diagnosis of gastroscopic biopsy via deep-learned single-shot femtosecond stimulated Raman histology
Source: Nat Commun. 2022 Jul 13;13:4050. doi: 10.1038/s41467-022-31339-8 (PMC9279377; doi:10.1038/s41467-022-31339-8)

Instant diagnosis of gastroscopic biopsy via deep-learned single-shot femtosecond stimulated Raman histology

Zhijie Liu[1#], Wei Su[2#], Jianpeng Ao[1#], Min Wang[3#], Qiuli Jiang[4], Jie He[4], Hua Gao[4], Shu Lei[5], Jinshan Nie[6], Xuefeng Yan[7], Xiaojing Guo[8], Pinghong Zhou[2*], Hao Hu[2,9*], Minbiao Ji[1*]

[1] State Key Laboratory of Surface Physics and Department of Physics, Human Phenome Institute, Academy for Engineering and Technology, Key Laboratory of Micro and Nano Photonic Structures (Ministry of Education), Yiwu Research Institute, Fudan University, Shanghai 200433, China

[2] Endoscopy Center and Endoscopy Research Institute, Zhongshan Hospital, Fudan University, Shanghai, 200032 China

[3] Department of Gastroenterology, Shanghai Children Hospital, Shanghai Jiaotong University, Shanghai, 200062, China

[4] Department of Pathology, Endoscopic Center, Zhongshan Hospital (Xiamen Branch), Fudan University, Xiamen, 361015, China

[5] Department of Gastroenterology, the Central Hospital of Wuhan, Wuhan, 430014, China

[6] Department of Gastroenterology, the 1st People's Hospital of Taicang, Soochow University, Suzhou, 215400, China

[7] Department of Gastroenterology, Shangrao Municipal Hospital, Shangrao, Jiangxi, 334000, China

[8] Department of Health Statistics, Second Military Medical University, Shanghai, China

[9] Department of Gastroenterology, People's Hospital of Shigatse, Shigatse, 857007, China

[#] These authors contributed equally.

***CORRESPONDENCE:** minbiaoj@fudan.edu.cn; hu.hao1@zs-hospital.sh.cn; zhou.pinghong@zs-hospital.sh.cn

Supplemental Materials

Table S1. Clinicopathologic characteristics of gastric lesions (JCGC, 3rd) of the 279 studied patient cases.

	Gastric cancer（n=103）	Non-cancerous lesion (n=176)
**Average age (SD)**	41.13 (±10.55）	32.12 (±14.59)
**Gender**		
Male	77 (65.0)	72 (40.9)
Female	36 (35.0)	104 (59.1)
**Location**		
Up 1/3	24 (23.3)	3 (1.7)
Middle 1/3	28 (27.2)	2 (1.2)
**Mean diameter (range)**	3.814	
**Macroscopic type**		
Type 0	18 (17.5)	NA
Type 1	19 (18.4)	NA
Type 2	46 (43.7)	NA
Type 3	10 (9.7)	NA
Type 4	9 (8.7)	NA
Type 5	2 (2.0)	NA
**Histological type**		
Noncancerous	NA	176 (100)
Papillary (pap)	3 (2.9)	NA
Tubular (tub)	43 (41.7)	NA
Poorly differentiated (por)	39 (37.9)	NA
Mucinous (muc)	6 (5.8)	NA
Signet ring cell (sig)	12 (11.7)	NA
**Degree of differentiation**		
Differentiated	46 (44.7)	NA
Undifferentiated	57 (55.3)	NA

Table S2. Number of included patients and images.

	Total images (patients)	Non-cancerous images (patient)	Cancer images (patients)
Training-Validation	62431(224)	41352(133)	21079(91)
Test	26756(55)	17722(43)	9034(12)
Total	89187(279)	59074(176)	30113(103)

	Total images (patients)	Differentiated images (patient)	Undifferentiated images (patients)
Training-Validation	16104(78)	10997(28)	5107(50)
Test	6902(17)	4713(13)	2189(4)
Total	23006(95)	15710(41)	7296(54)

Table S3. Diagnostic comparison between pathologists and CNN based femto-SRH. Two-side statistical tests were conducted and the *p*-value was regarded as statistical significance.

		McNemar test (P value)
Cancerous Identification	SRH vs P1	1
	SRH vs P2	1
	SRH vs P3	0.125
	SRH vs P4	0.125
Differentiation Determination	SRH vs P1	0.625
	SRH vs P2	1
	SRH vs P3	1
	SRH vs P4	0.063

Fig. S1. Optical lay-out of the femto/pico dual-mode SRS microscope. The dash-shaded area represents the femto-SRS part with transform limited femtosecond pulses, while the paths going through SF57 glass rods chirped the pulses to picoseconds for pico-SRS. EOM: electro-optical modulator; PD: photodiode; PMT: photo multiplier tube; LIA: lock-in amplifier; DM: dichroic mirror; GM: galvo mirror.

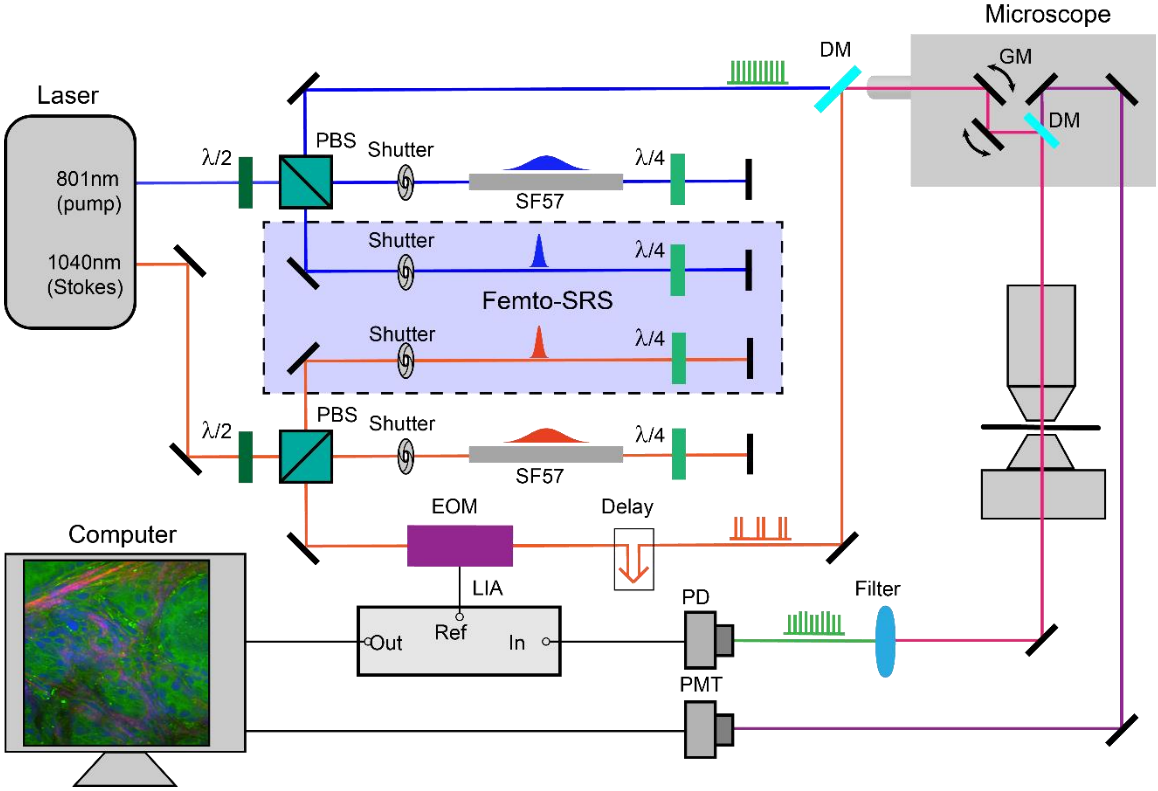

Fig. S2. U-Net conversion of a typical gastric tissue specimen. (a) The prediction results from a single raw femto-SRS image to the dual-channel images, in comparison to the ground truth. (b) Line cut intensity to illustrate the prediction accuracy. Scale bars: 50 μm. Figures are representative of five independent experiments. Source data are provided in the Source data file.

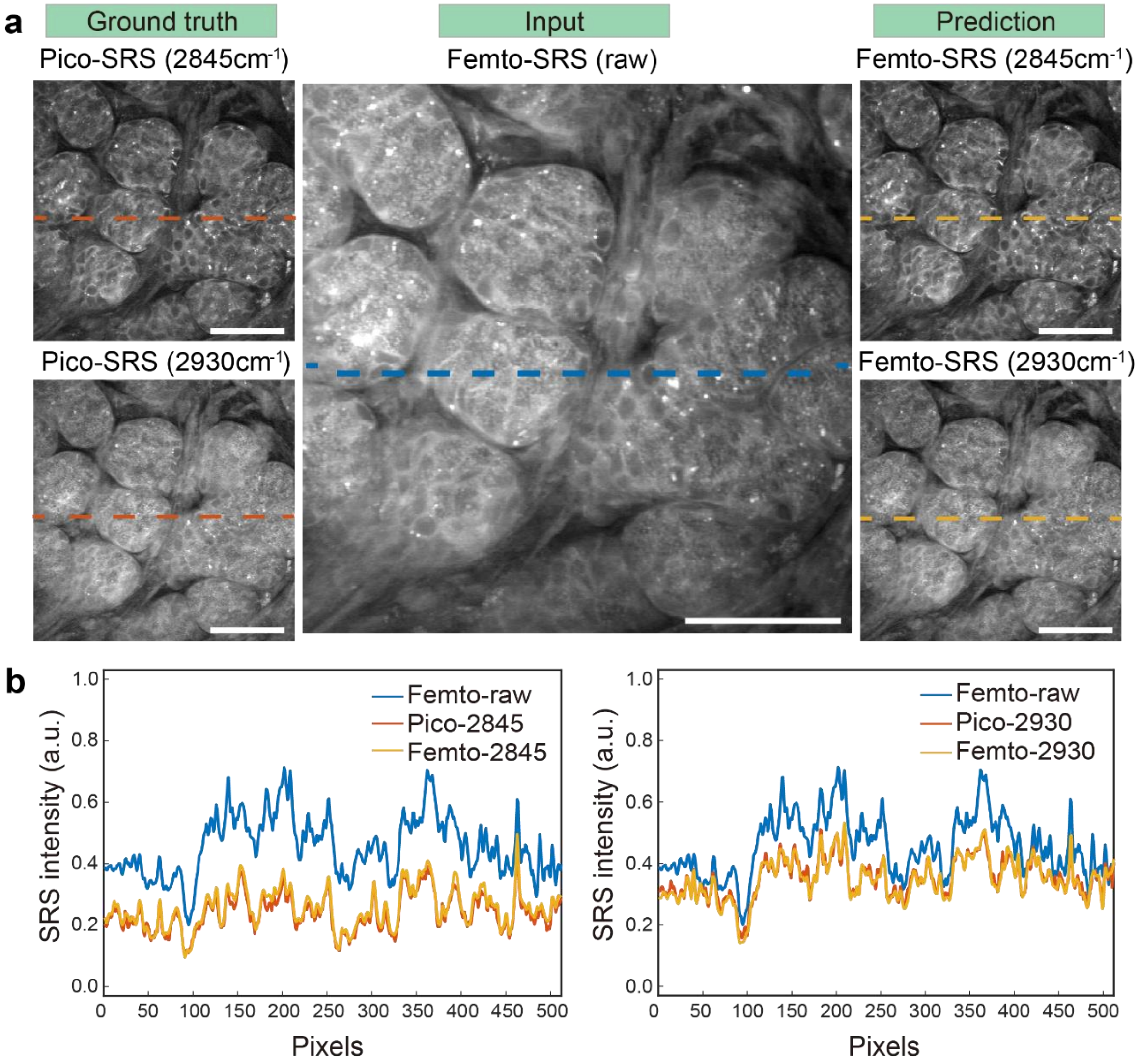

Fig. S3. Representative histological features revealed by femto-SRS images of fresh gastric tissues. (a-e) Normal gastric glands imaged in different sections and states. (f) Intestinal metaplasia with cup-shaped cells. (g-h) Moderately and poorly differentiated adenocarcinoma. Scale bars: 50 μm. Figures are representative of twenty independent experiments.

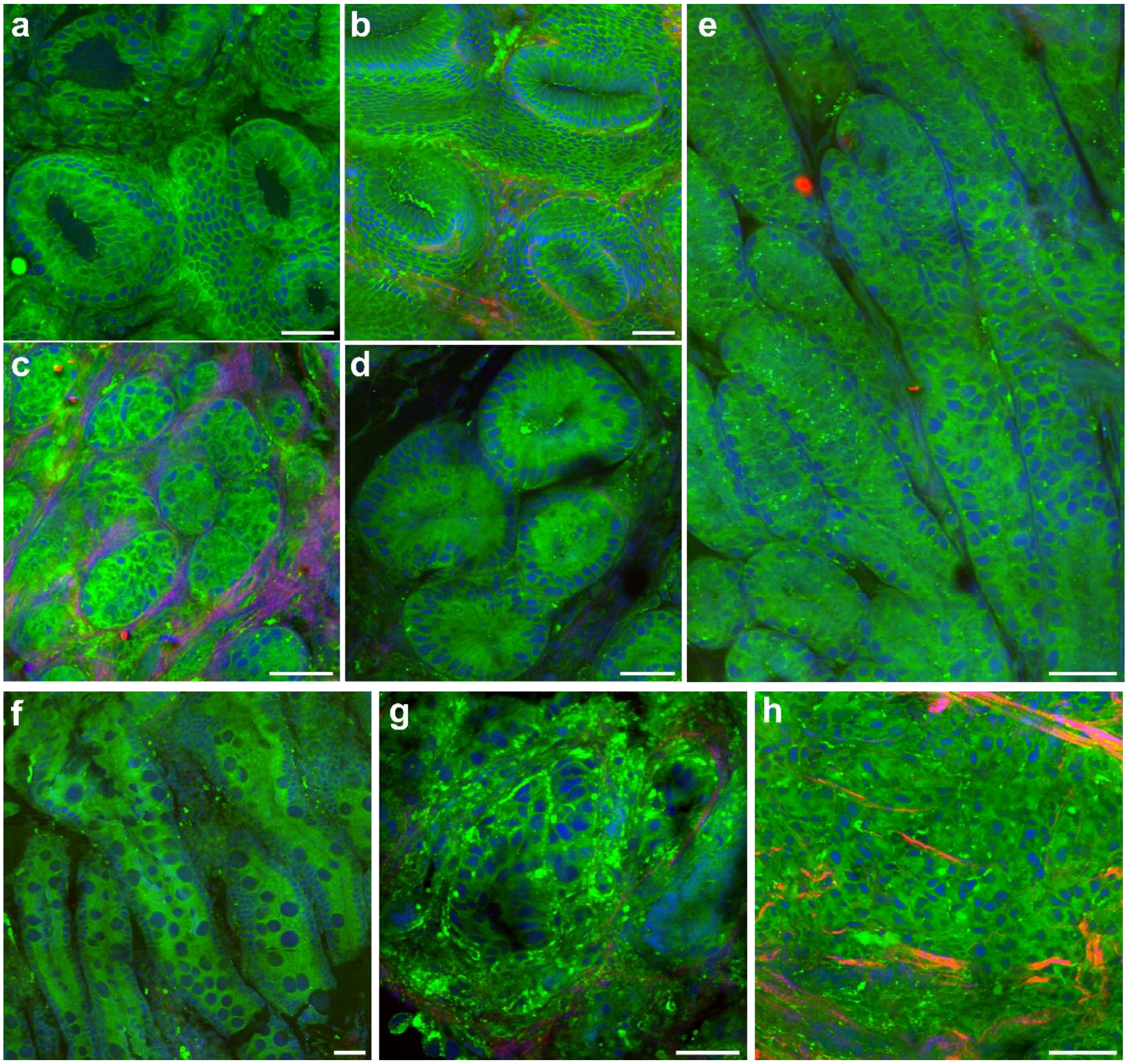

Fig. S4. Work flow of the imaging, CNN training, validation and prediction of large femto-SRS images.

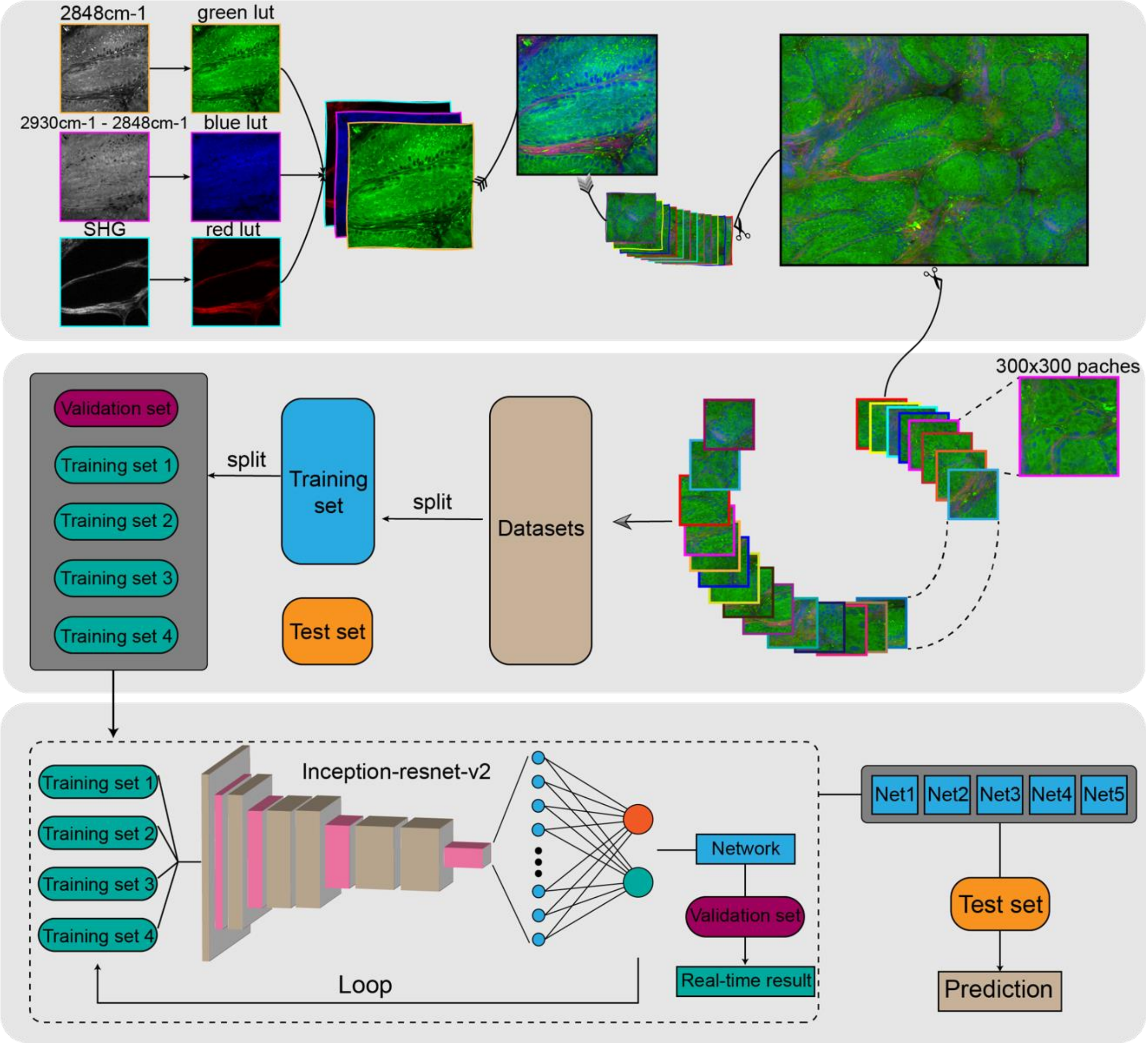

Fig. S5. Training results of the CNNs used to classify (a) cancer vs. non-cancer, and (b) differentiated vs. undifferentiated cancers. The loss function, accuracy function and ROC curves showed the convergency and performance of the neural networks. The Youden's index was found from the ROC curve as the cut-off point for predicting test dataset. Source data are provided in the Source data file.

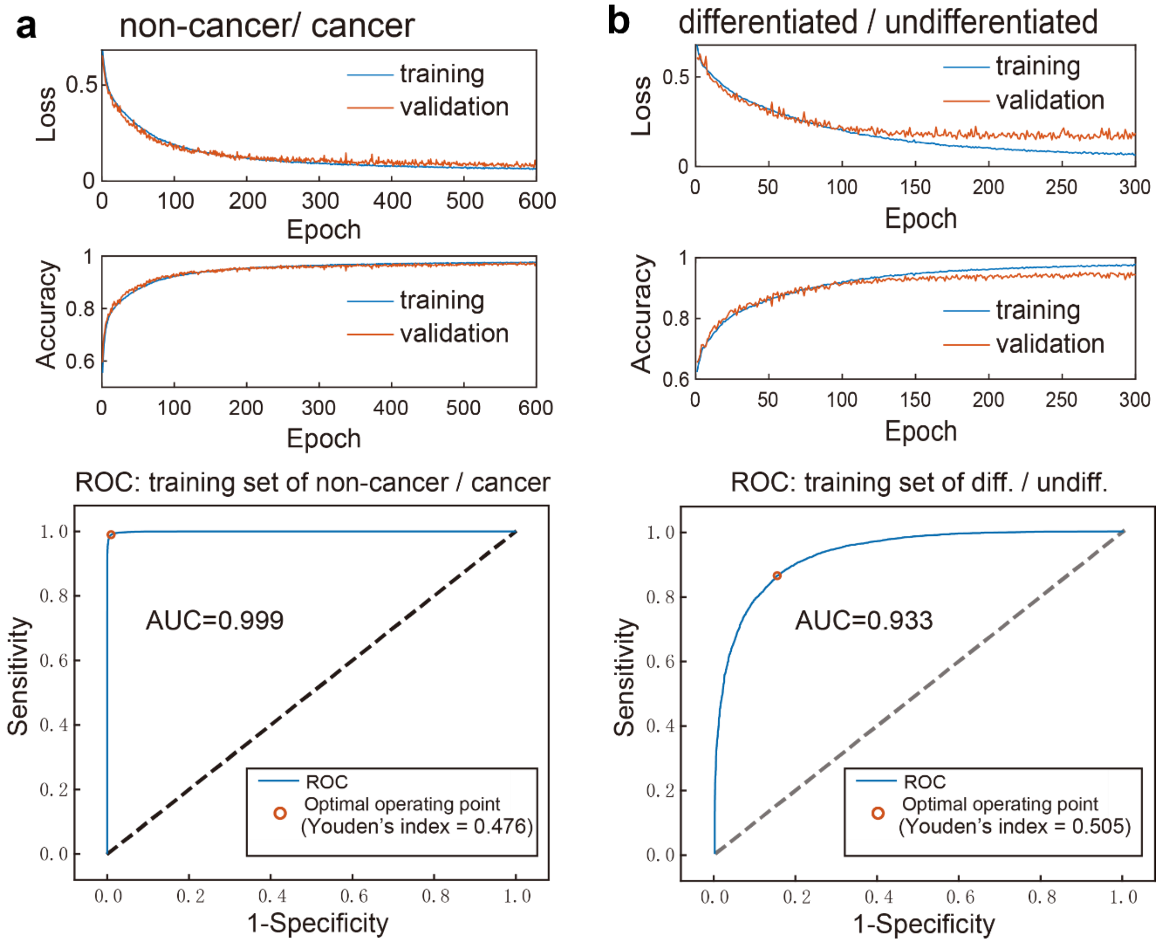

Fig. S6. Diagnostic prediction results of CNN based femto-SRH, compared with the four pathologists on test cases. (a) Non-cancer vs. cancer; (b) Differentiated vs. undifferentiated cancers.

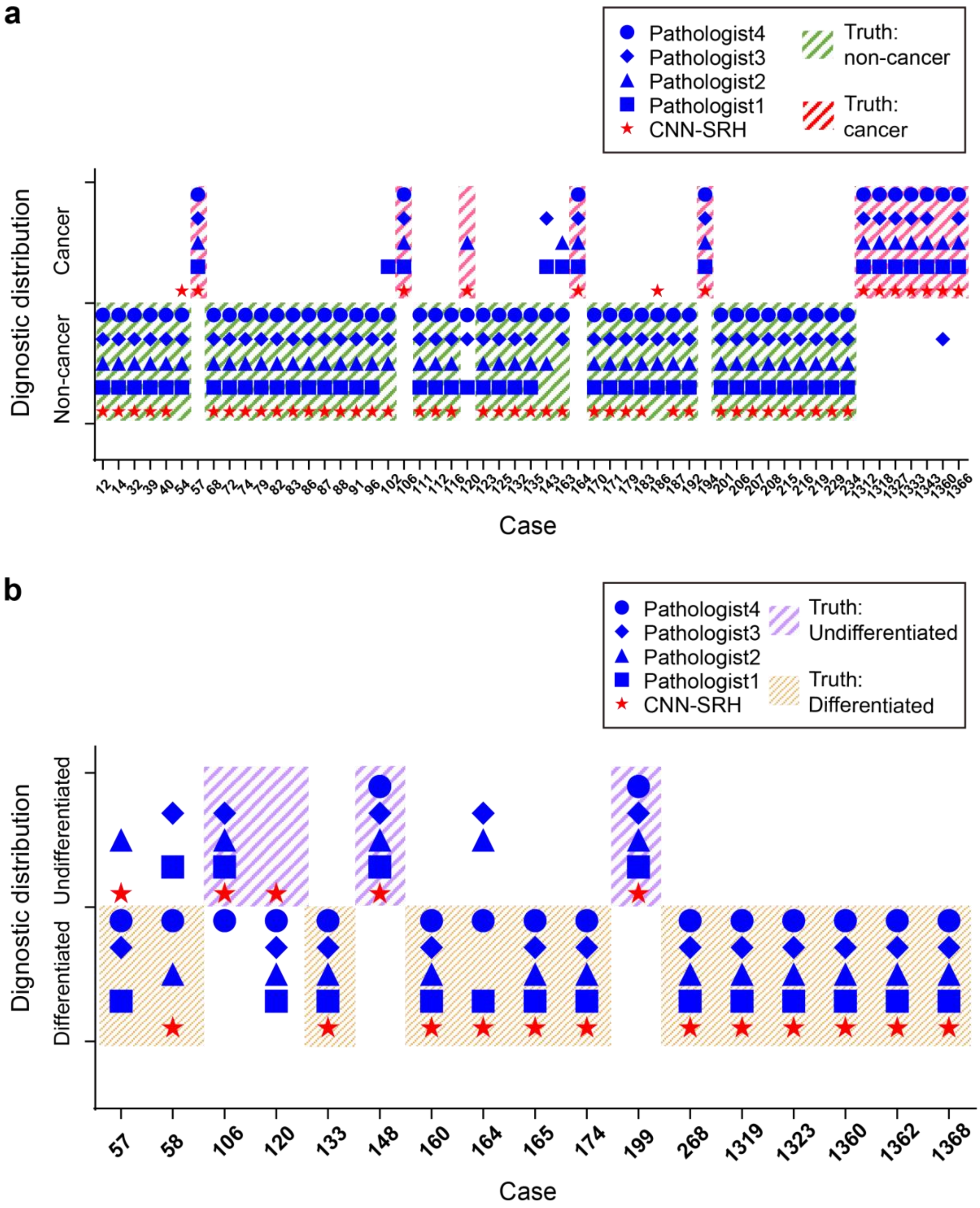

Fig. S7. Workflow of the semantic segmentation of CNN. (a-c) Flip-expansion of a large femto-SRS image, followed by predictions on shifting small tiles. (d) Classification workflow. (e) Segmentation and (f) heatmap coloring. Scale bars: 100 µm. Source data are provided in the Source data file.

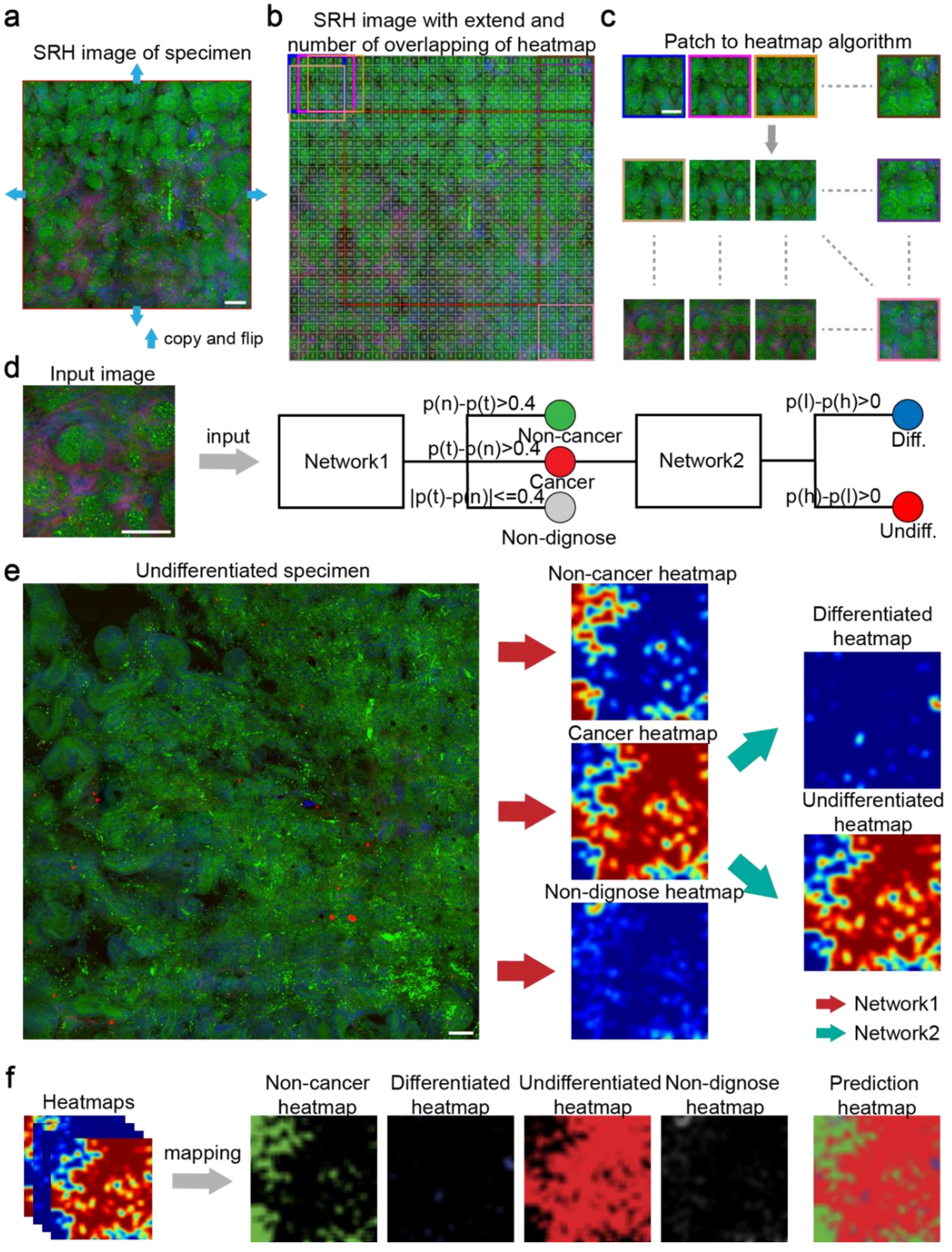

Fig. S8. Demonstration of the two misclassified non-cancer cases. (a) case #54 with degraded tissues; (b) case #186 with dense macrophage-like cells in inflammatory tissue. Red squares: predicted cancer: cyan squares: predicted non-cancer. Source data are provided in the Source data file.

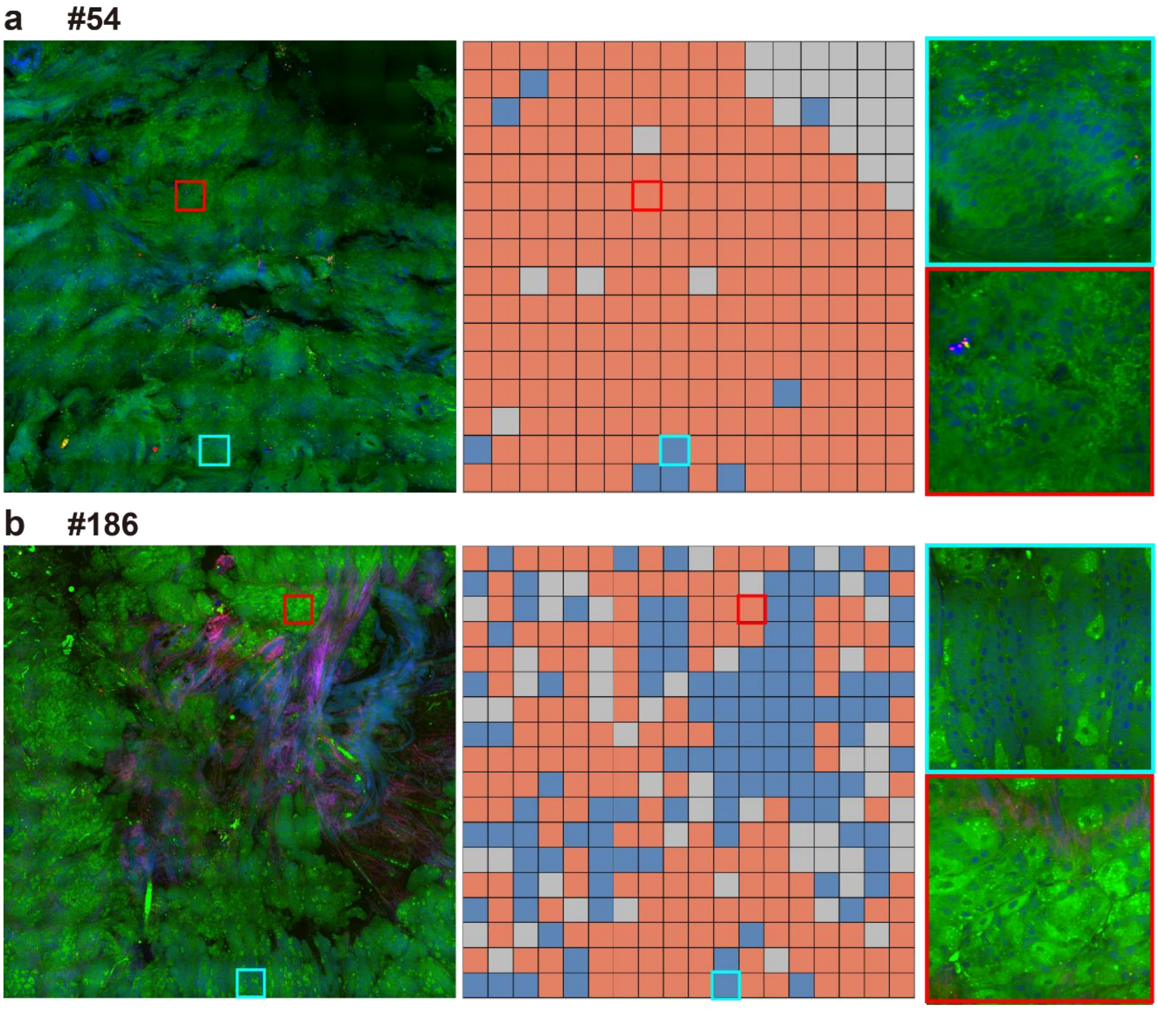

Fig. S9. Histograms of quantitative biochemical compositions of all the gastric biopsy cases and in the subgroups of non-cancer and cancer cases. (a) Lipid/protein ratio; (b) Collagen area ratio. Source data are provided in the Source data file.

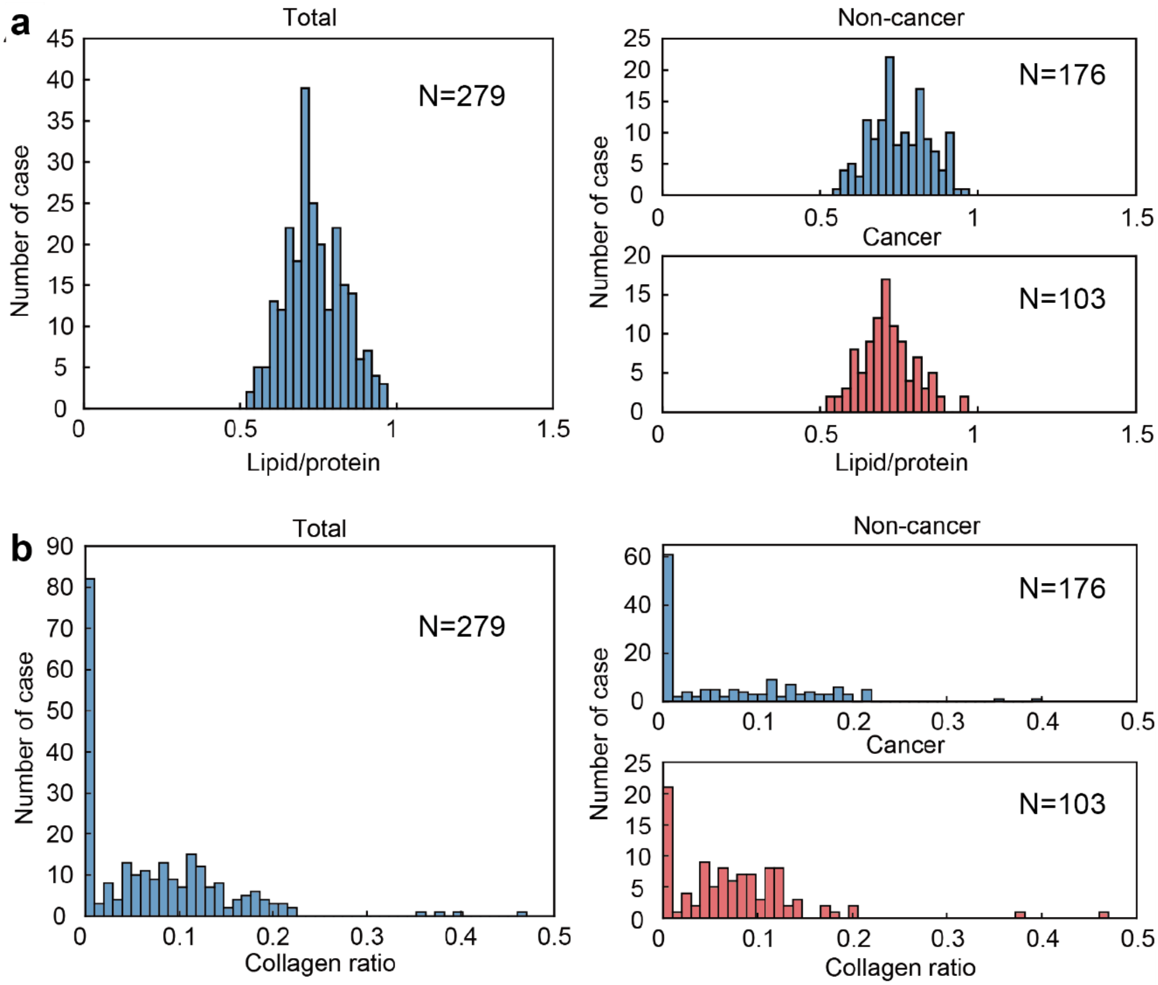

Supplement: Supplementary file 1 — Supplementary Information [file 41467_2022_31339_MOESM1_ESM.pdf]
