# Supplementary material for: Instant diagnosis of gastroscopic biopsy via deep-learned single-shot femtosecond stimulated Raman histology
Source: Nat Commun. 2022 Jul 13;13:4050. doi: 10.1038/s41467-022-31339-8 (PMC9279377; doi:10.1038/s41467-022-31339-8)
Supplement: Supplementary file 2 — Description of Additional Supplementary Files [file 41467_2022_31339_MOESM2_ESM.pdf]

Description of Additional Supplementary Files

File Name: Supplementary Movie 1
Description: A representative SRS image of normal gastroscopic biopsy.

File Name: Supplementary Movie 2
Description: A representative SRS image of cancerous gastroscopic biopsy.
