# Supplementary material for: Instant diagnosis of gastroscopic biopsy via deep-learned single-shot femtosecond stimulated Raman histology
Source: Nat Commun. 2022 Jul 13;13:4050. doi: 10.1038/s41467-022-31339-8 (PMC9279377; doi:10.1038/s41467-022-31339-8)
Supplement: Supplementary file 3 — Reporting Summary [file 41467_2022_31339_MOESM3_ESM.pdf]

nature portfolio

Corresponding author(s): Minbiao Ji, Hao Hu, Pinghong Zhou

Last updated by author(s): May 25, 2022

Reporting Summary

Nature Portfolio wishes to improve the reproducibility of the work that we publish. This form provides structure for consistency and transparency in reporting. For further information on Nature Portfolio policies, see our Editorial Policies and the Editorial Policy Checklist.

Statistics

For all statistical analyses, confirm that the following items are present in the figure legend, table legend, main text, or Methods section.

n/a	Confirmed	
☐	☒	The exact sample size (*n*) for each experimental group/condition, given as a discrete number and unit of measurement
☐	☒	A statement on whether measurements were taken from distinct samples or whether the same sample was measured repeatedly
☐	☒	The statistical test(s) used AND whether they are one- or two-sided<br>*Only common tests should be described solely by name; describe more complex techniques in the Methods section.*
☒	☐	A description of all covariates tested
☒	☐	A description of any assumptions or corrections, such as tests of normality and adjustment for multiple comparisons
☐	☒	A full description of the statistical parameters including central tendency (e.g. means) or other basic estimates (e.g. regression coefficient) AND variation (e.g. standard deviation) or associated estimates of uncertainty (e.g. confidence intervals)
☐	☒	For null hypothesis testing, the test statistic (e.g. *F*, *t*, *r*) with confidence intervals, effect sizes, degrees of freedom and *P* value noted<br>*Give P values as exact values whenever suitable.*
☒	☐	For Bayesian analysis, information on the choice of priors and Markov chain Monte Carlo settings
☐	☒	For hierarchical and complex designs, identification of the appropriate level for tests and full reporting of outcomes
☐	☒	Estimates of effect sizes (e.g. Cohen's *d*, Pearson's *r*), indicating how they were calculated

Our web collection on statistics for biologists contains articles on many of the points above.

Software and code

Policy information about availability of computer code

Data collection	SRS images were collected with homebuilt system composed with Spectra Physics Insight DS+ and Olympus FV1200.
Data analysis	ImageJ 1.51j8(https://imagej.nih.gov/ij/) was used to colored and analysis the raw SRS images; Matlab 2021a(https://ww2.mathworks.cn/) and Python 3.8.5(https://www.python.org/) were used to process data and perform deep learning; torch 1.7.0, torchvision 0.8.1, numpy 1.19.2, pandas 1.1.3, PIL 8.0.1, opencv 4.4.0 were used to design the deep learning algorithm. All the codes are available at (https://zenodo.org/record/6582765). Statistical analyses were run on SPSS (version 9.0) and R software for Windows (version 3.5.1; http://www.r-project.org).

For manuscripts utilizing custom algorithms or software that are central to the research but not yet described in published literature, software must be made available to editors and reviewers. We strongly encourage code deposition in a community repository (e.g. GitHub). See the Nature Portfolio guidelines for submitting code & software for further information.

Data

Policy information about availability of data

All manuscripts must include a data availability statement. This statement should provide the following information, where applicable:

- Accession codes, unique identifiers, or web links for publicly available datasets
- A description of any restrictions on data availability
- For clinical datasets or third party data, please ensure that the statement adheres to our policy

The data that support the findings of this study are provided in Supplementary Videos S1-S2, Supplementary Tables S1-S3 and Supplementary Figures S1-S9. Data used for training and test the deep-learning models are available under accession code [https://zenodo.org/record/6582765]. Remaining data are available from the corresponding author upon request. All data access in this study are restricted for scientific research purpose only. Source data are provided with this paper.

Field-specific reporting

Please select the one below that is the best fit for your research. If you are not sure, read the appropriate sections before making your selection.

☒ Life sciences ☐ Behavioural & social sciences ☐ Ecological, evolutionary & environmental sciences

For a reference copy of the document with all sections, see nature.com/documents/nr-reporting-summary-flat.pdf

Life sciences study design

All studies must disclose on these points even when the disclosure is negative.

Sample size	For Femto-SRS projection via U-Net, we chose the sample size based on whether the trained network can project the single-shot Femto-SRS images to dual-channel pico-SRS images in the testing set with preserved spatio-chemical information. The quality is quantitatively evaluated by comparing the intensity profiles along the line-cuts between the ground truth image and the projected image. Totally, 50 pairs of femto/pico-SRS images of HeLa cells and 100 pairs of gastric tissues were used.<br>For CNN demonstration, gastroscopic biopsies from 279 patients were used in the training and test of the model, which was determined with the diagnostic performance of the network output.
Data exclusions	No data were excluded from the analyses.
Replication	The work was applied in various experiments. Multiple experiments were repeated with similar results as reported in the Methods and figure legends.
Randomization	For U-Net, the training set was generated by randomly selecting a set of Femto-SRS and pico-SRS image pairs of cells and tissues. For CNN, the training set was randomly selected from 279 cases. The testing set was randomly selected which was not involved in the training.
Blinding	For both demonstration, the training and testing set was randomly allocated with no overlapping. The training and optimization of both networks (U-Net and CNN) were solely based on the training set, the performance was evaluated by the testing set which was kept independent from the training process.

Reporting for specific materials, systems and methods

We require information from authors about some types of materials, experimental systems and methods used in many studies. Here, indicate whether each material, system or method listed is relevant to your study. If you are not sure if a list item applies to your research, read the appropriate section before selecting a response.

Materials & experimental systems

n/a	Involved in the study	
☒	☐	Antibodies
☐	☒	Eukaryotic cell lines
☒	☐	Palaeontology and archaeology
☒	☐	Animals and other organisms
☐	☒	Human research participants
☒	☐	Clinical data
☒	☐	Dual use research of concern

Methods

n/a	Involved in the study	
☒	☐	ChIP-seq
☒	☐	Flow cytometry
☒	☐	MRI-based neuroimaging

Eukaryotic cell lines

Policy information about cell lines

Cell line source(s)	HeLa cell line was purchased from ATCC.
Authentication	Not authenticated.
Mycoplasma contamination	The HeLa cell line was not tested for mycoplasma contamination.
Commonly misidentified lines<br>(See ICLAC register)	None

Human research participants

Policy information about studies involving human research participants

Population characteristics	The population characteristics was detailed in Table S1, which could be briefly summarized below:

Population characteristics	Total 279 patients included 103 gastric cancer cases and 179 non-cancerous lesion cases. Among the cancerous class, the average age is 41.13 and most patients are male (n=77). The average age of patients in non-cancerous class is 32.12, which containing 72 male and 104 female cases.
Recruitment	The patients were recruited if they met the criteria: (1)18-70 years of age; (2) American Society of Anesthesiologists (ASA) class 1–3; (3) single stomach lesion. The exclusion criteria were: (1) history of allergies; (2) previous abdominal surgery; (3) pregnant woman.<br>Fresh gastroscopic biopsies were taken by endoscopic forceps (Alton, AF-D2416BTC, China) with typical size of ~ 2 × 2 mm2. Surgical tissues from endoscopic submucosal dissection (ESD) specimens were obtained with an approximate size of 2 × 2 mm2.
Ethics oversight	The study was conducted in accordance with the Declaration of Helsinki, and the protocol was approved by the Ethics Review Board of the Zhongshan hospital (B2021-122R2). Written informed consent was obtained from each patient.

Note that full information on the approval of the study protocol must also be provided in the manuscript.
